# Association between immune-inflammatory index and osteoporosis: a systematic review and meta-analysis

**DOI:** 10.1186/s40001-025-02893-w

**Published:** 2025-07-16

**Authors:** Bihui Bai, Xingwen Xie, Yongyu Yue, Jiahe Cui, Fangfang Xie, Fei Yao

**Affiliations:** 1 Shanghai Municipal Hospital of Traditional Chinese Medicine, Shanghai University of Traditional Chinese Medicine, Shanghai, China; 2 Gansu Provincial Hospital of Traditional Chinese Medicine, Gansu, China; 3 Affiliated Hospital of Gansu University of Chinese Medicine, Lanzhou, China; 4 School of Acupuncture-Moxibustion and Tuina, Shanghai University of Traditional Chinese Medicine, Shanghai, China

**Keywords:** Osteoporosis, Immune-inflammatory index, Systematic review, Meta-analysis

## Abstract

**Objective:**

Plenty of evidence proves the prospective diagnostic and prognostic utility of inflammatory markers in osteoporosis (OP). However, the relations of immune-inflammatory indices to OP remain elusive, with scarce conclusive evidence-based findings. Our systematic review and meta-analysis endeavored to unveil the links between immune-inflammatory indices and OP.

**Methods:**

PubMed, Web of Science, Embase, as well as Cochrane Library, were thoroughly retrieved for research investigating the links of immune-inflammatory indices to OP, from database inception to February 20, 2025. Data were analyzed using odds ratio (OR), standardized mean difference (SMD), as well as corresponding 95% confidence interval (CI). Sensitivity and subgroup analyses were carried out for result robustness evaluation and heterogeneity source identification. Review Manager 5.4 and STATA 18.0 were utilized in every statistical analysis.

**Results:**

A total of 24 studies were included in this analysis. Eight cohort studies and 16 case–control studies based on 397,525 subjects and 11,904 cases were eventually screened and retained. The findings indicated significant positive relations of immune-inflammatory indices to OP risk. For categorical variables, elevated neutrophil-to-lymphocyte ratio (NLR) (OR = 2.34, 95% CI 1.77–3.11; *P* < 0.00001), platelet-to-lymphocyte ratio (PLR) (OR = 1.05, 95% CI 1.01–1.08; *P* = 0.01), as well as systemic immune-inflammation index (SII) (OR = 1.16, 95% CI 1.04–1.30; *P* = 0.01) notably correlated with a increased OP risk. For continuous variables, individuals with OP exhibited significantly higher levels of NLR (SMD = 0.71, 95% CI 0.35–1.07; *P* = 0.0001), PLR (SMD = 0.43, 95% CI 0.17–0.68; *P* = 0.001), monocyte-to-lymphocyte ratio (MLR) (SMD = 0.54, 95% CI 0.16–0.91; *P* = 0.005), and SII (SMD = 0.25, 95% CI 0.03–0.47; *P* = 0.03) compared to non-OP populations. Subgroup analyses revealed that geographic region and age were major contributing factors influencing the association between immune-inflammatory indices and OP.

**Conclusion:**

Immune-inflammatory indices such as NLR, PLR, MLR, and SII are significantly linked to increased risk of OP. These indices may facilitate the early identification of individuals at high risk for OP and support timely preventive strategies. Given the inherent limitations of the current study, further prospective, multicenter clinical investigations are warranted to validate the relations of immune-inflammatory indices to OP.

*Trial registration* Systematic review registration: PROSPERO, identifier: CRD420250656296.

**Supplementary Information:**

The online version contains supplementary material available at 10.1186/s40001-025-02893-w.

## Introduction

OP is a systemic bone disease featuring decreased bone density and bone microstructure degradation, causing more fragile bone and an increased likelihood of fractures [1]. Clinically, OP presents with significant manifestations and, in the context of an aging global population, is among the most common and frequently occurring chronic diseases globally [2, 3]. The pathogenesis of OP is multifactorial, and its treatment is typically prolonged, often yielding slow and suboptimal therapeutic outcomes. In severe cases, patients may experience fractures, which further compromise their physical and psychological well-being, ultimately diminishing their quality of life. According to national demographic statistics in China [4], there are approximately 210 million individuals aged 60 or older, making it the country with the most old people globally. With the continual rise in population and average life expectancy, the burden posed by OP has become increasingly prominent. Therefore, in addition to active clinical diagnosis and treatment, the quality of life in middle-aged and old populations should be prioritized during the progression to OP. Timely and effective health interventions are essential to reducing both the incidence and the linked adverse outcomes of OP.

OP is a multifactorial condition influenced by endocrine, metabolic, and mechanical factors. Inflammation is critical in bone metabolism, directly impacting bone growth and development, and contributing to alterations in bone resorption. Dysregulation of the immune-inflammatory system can result in sustained bone destruction. This is particularly evident among postmenopausal females, often displaying a sustained mild inflammatory state accompanied by alterations in cytokine patterns and shifts in immune cell populations [5]. In recent years, new inflammatory markers such as the neutrophil-to-lymphocyte ratio (NLR), platelet-to-lymphocyte ratio (PLR), monocyte-to-lymphocyte ratio (MLR), systemic immune-inflammation index (SII), systemic inflammatory response index (SIRI), and pan-immune-inflammation value (PIV) have gained considerable attention for their potential diagnostic and prognostic value in OP [6]. In the field of public health, low-cost and efficient screening methods for OP are of paramount significance. Routine complete blood count (CBC) testing represents an ideal screening approach due to its low cost and ease of implementation, rendering it highly valuable for widespread clinical application.

Before the present study, only two meta-analyses investigated the links of immune-inflammatory indices to OP. One study [7] investigated the connections of NLR and PLR with OP, including 10 eligible articles published before April 2022. The other [8] focused exclusively on postmenopausal women, analyzing 8 studies published before June 2022, and examined only the association between NLR and OP. While these analyses provided preliminary evidence supporting the potential role of NLR and PLR in OP, their limited sample sizes and scope restrict the generalizability and comprehensiveness of the conclusions. With more literature since 2022, additional studies have been published exploring a broader array of inflammatory indices, including MLR, SII, SIRI, and PIV, for a better understanding of systemic immune-inflammatory status in OP. These emerging indices not only expand the scope of the investigation, but also offer novel insights into the mechanisms and potential clinical interventions for OP. Therefore, the present meta-analysis endeavors to evaluate the links of various immune-inflammatory indices to OP, providing quantitative and evidence-based insights into their potential predictive value for OP risk.

## Materials and methods

This study was conducted under the Preferred Reporting Items for Systematic Reviews and Meta-Analyses (PRISMA) guidelines [9] and registered in the PROSPERO database (Registration No. CRD420250656296).

### Literature retrieval and selection

Two investigators (FFX and YHY) jointly developed the search strategy. They independently constructed a comprehensive search across PubMed, Embase, Web of Science, as well as Cochrane Library from establishment to February 20, 2025 utilizing Medical Subject Headings (MeSH) and free-text keywords: “Lymphocytes”, “Age-related OP”, “Lymphocyte”, “Lymphoid Cells”, “Lymphoid Cell”, “Ratio”, “OP”, “Age-Related Osteoporoses”, “Age-Related OP”, “Age-Related Bone Loss”, “Age-Related Bone Losses”, “Senile Osteoporoses”, “Senile OP”, “Post-Traumatic Osteoporoses”, and “Post-Traumatic OP”. The strategy is detailed in Supplementary Table S1.

### Eligibility criteria

Inclusion criteria were: (1) published and complete cohort studies, case–control studies, or clinical trials; (2) study participants were diagnosed with OP based on the World Health Organization (WHO) diagnostic criteria [10], which involve bone mineral density (BMD) assessment by dual-energy X-ray absorptiometry (DXA) and T-score classification: normal (T-score ≥ − 1.0), osteopenia (T-score < − 1.0 to − 2.5), and OP (T-score ≤ − 2.5); eligible participants included OP patients of any sex, age, or nationality; (3) at least one immune-inflammatory index associated with OP was reported, including NLR, PLR, lymphocyte-to-monocyte ratio (LMR), SII, SIRI, and PIV; (4) sufficient data were available to calculate the odds ratio (OR) or standardized mean difference (SMD) and corresponding 95% confidence interval (CI) for the association between immune-inflammatory indices and OP; (5) the study was fully published.

Exclusion criteria were: (1) duplicate publications based on the same dataset; (2) literature types such as reviews, letters, commentaries, and conference abstracts; (3) studies lacking sufficient data to calculate ORs and 95% CIs; (4) studies that did not provide relevant data; (5) studies with duplicated or overlapping data.

Two investigators (BHB and XWX) independently screened titles and abstracts. Full texts of possibly eligible articles were checked. Any dissents arising in the process were addressed after discussion and consensus.

### Data extraction

Data were extracted independently by two investigators (YHY and CJH), with dissents settled through consensus among all co-authors. A Microsoft Excel spreadsheet was used to record key information: first author, publication year, country, study type, sample size, mean age, follow-up duration, ORs, SMDs, and 95% CIs for various immune-inflammatory indices. Notably, in studies reporting LMR data. To convert LMR-related results into MLR format for compatibility with our statistical analysis framework, mathematical transformations were performed on the original OR values and their corresponding CIs. Specifically, the reciprocal of the reported OR and its confidence limits was taken, with the upper and lower bounds interchanged. This conversion preserved statistical logical consistency and ensured compatibility with standard analytical models involving MLR, thereby enhancing the interpretability and applicability of the data.

### Quality assessment

The study quality was rated independently by two investigators (YHY and CJH) via the Newcastle–Ottawa Scale (NOS). Any disagreements were adjudicated by a third senior reviewer (FY). The NOS assesses studies across selection, comparability, and outcome. The highest score was 9. A score of 7–9 denoted high quality [11].

### Statistical analysis

Meta-analysis was enabled by STATA 18.0 and Review Manager 5.4. The primary outcomes were the links of immune-inflammatory indices to OP, reported as both categorical and continuous variables. Pooled ORs, SMDs, and 95% CIs were computed. Heterogeneity across studies was detected via Cochran’s *Q* test (Chi2test) and the Higgins *I*^2^ statistic. *I*^2^ > 50% or *P* < 0.1 indicated significant heterogeneity. In such cases, a random-effects (DerSimonian–Laird) model was leveraged. To evaluate the robustness of our results and identify possible sources of heterogeneity, sensitivity and subgroup analyses were undertaken. Funnel plots and Egger’s regression test were utilized for publication bias detection, with *P* < 0.05 suggesting statistical significance.

## Results

### Literature search results

**Fig. 1:**
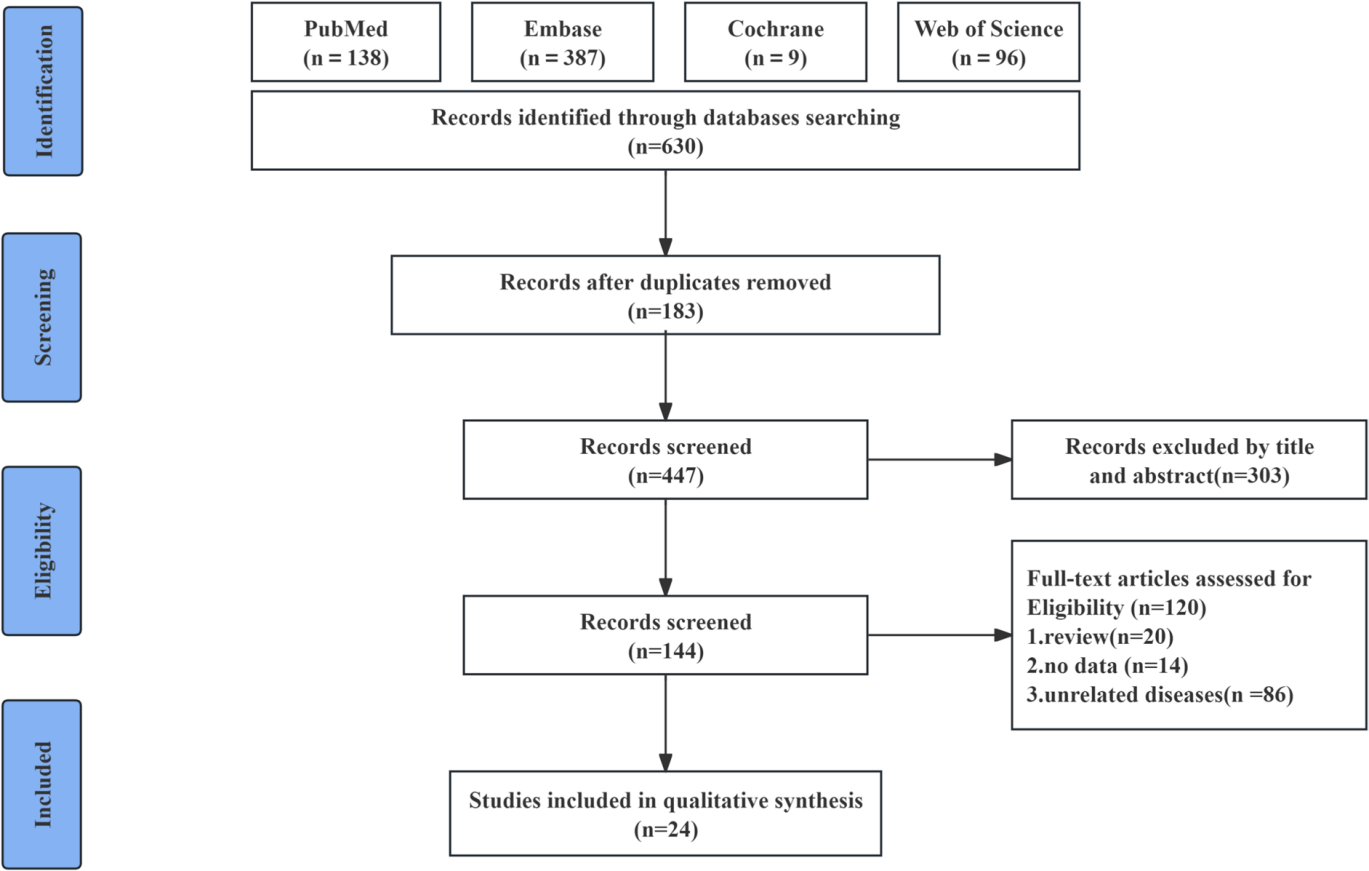
Flowchart of study screening and selection process

630 articles were retrieved at first. 183 duplicates were removed, and 303 were ostracized after title and abstract review. Among the left 144 articles, 20 were reviews, 14 lacked extractable data, and 86 were excluded because of irrelevance to the disease of interest. At last, 24 studies were incorporated for our quantitative analysis. The process and results are presented in Fig. 1.

### Characteristics of included studies

The 24 eligible articles were published between 2013 and 2024, with research conducted in the following regions: China [12, 13, 18–22, 24, 26, 28, 32, 33], Britain [14], America [15], South Korea [16], Turkey [17, 23, 25, 29–31, 34, 35], and the Sultanate of Oman [27]. Among them, 8 were cohort studies and 16 were case–control studies, involving 397,525 participants and 11,904 cases. The average age across all studies was 58.44; the mean age in cohort studies was 60.02, while that of case–control studies was 58.07. The general characteristics are displayed in Table 1.

**Table 1:** Basic characteristics of the included literature

Author	Study period	Region	Study design	Population	Sample size	Gender (male/female)	Mean age (years)	Markers	Quality score
Zhang [12]	2021.1–2022.12	China	Cohort	Postmenopausal women	966	0/966	61	MLR,NLR, PLR, SII	8
Ma [13]	2021.1–2023.12	China	Cohort	Elderly hypertensive patients	856	331/525	67.19 ± 6.17	SIRI	9
Dong [14]	2006–2010	UK Biobank	Cohort	Systemic inflammation patients	393,443	189,757/203686	56.05 ± 8.08	PLR, NLR, SII	7
Tang [15]	2007–2018	United States	Cohort	Postmenopausal women	893	0/893	60.90 ± 0.26	NLR, PLR, SII	7
Song [16]	2005–2017	Korea	Cohort	Postmenopausal women with rheumatoid arthritis	613	0/613	61.15	MLR, PLR,NLR	7
Karatas [17]	2019.1–2019.7	Turkey	Cohort	Nondialysis CKD patients	283	163/120	66.9 ± 12.3	PLR, NLR,	7
Fang [18]	2015.1–2017.1	China	Cohort	Postmenopausal women	238	0/238	60.5 ± 8.1	SII, LMR, PLR, NLR	8
Huang [19]	2012.4–2015.9	China	Cohort	Postmenopausal women	233	0/233	57.9	NLR	7
Hang [20]	2022.1–2023.5	China	Case–control	Male	826	826/0	62.5	SII, NLR, LMR, PLR	7
Yan [21]	2020.1–2024.7	China	Case–control	Non-diabetic elderly	438	216/222	57.73	PLR, NLR, MLR, SII, SIRI, PIV	7
Yuan [22]	2022.1–2024.1	China	Case–control	Osteoporosis in postmenopausal type 2 diabetic patients	320	0/320	63.67	NLR, MLR, PLR	7
Busra [23]	2018.1–2023.12	Turkey	Case–control	Postmenopausal women	368	0/368	57.67	NLR, PLR, MLR, SII, SIRI, PIV	7
Zhang [24]	2005–2008	China	Case–control	Middle‐aged and older people	4625	2326/2299	62.55 ± 11.33	SII	8
Hakan [25]	2021.1–2022.1	Turkey	Case–control	Postmenopausal women	527	0/527	53.03	NLR, PLR, MLR, SII	8
Nie [26]	2018.1–2020.12	China	Case–control	Older Chinese people	648	232/416	67.21 ± 6.40	SIRI, NLR,AISI, PLR, LMR,	7
Asma [27]	2017.1–2019.12	Sultanate of Oman	Case–control	Postmenopausal women	450	0/450	63.69 ± 8.23	NLR, MLR, PLR,	7
Gao [28]	2015.1–2018.8	China	Case–control	Osteoporosis patient	292	122/170	56.46	MLR, NLR, PLR	7
Semra1 [29]	2017.10–2018.1	Turkey	Case–control	Postpartum women	93	0/93	29.57	PLR, MLR, NLR	7
Semra2 [30]	2016.7–2017.12	Turkey	Case–control	Postmenopausal women	252	0/252	54.6	PLR, NLR, MLR	7
Koseoglu [31]	2015.5–2016.5	Turkey	Case–control	Postmenopausal women	211	0/211	56.8	NLR, PLR	8
Liu [32]	2013.1–2014.12	China	Case–control	Postmenopausal women	269	0/269	52.5	NLR	7
Yu [33]	2009.1–2010.12	China	Case–control	Postmenopausal women	512	0/512	74.95	NLR	7
Yilmaz [34]	2008.1–2011.12	Turkey	Case–control	Postmenopausal women	438	0/438	64	NLR	7
Zeynel [35]	NA	Turkey	Case–control	Elderly people	1635	685/950	71.43	NLR	7

*NA* not available, *NLR* neutrophil-to-lymphocyte ratio, *PLR* platelet-to-lymphocyte ratio, *MLR* monocyte-to-lymphocyte ratio, *SII* systemic immune-inflammation index, *SIRI* systemic inflammatory response index, *PIV* pan-immune-inflammation value

### Study quality

All eligible studies scored 7–8 on the NOS, revealing relatively high methodological quality (Supplementary Tables S2 and S3).

### Meta-analysis results

#### Association between NLR and OP risk (categorical variable)

**Fig. 2:**
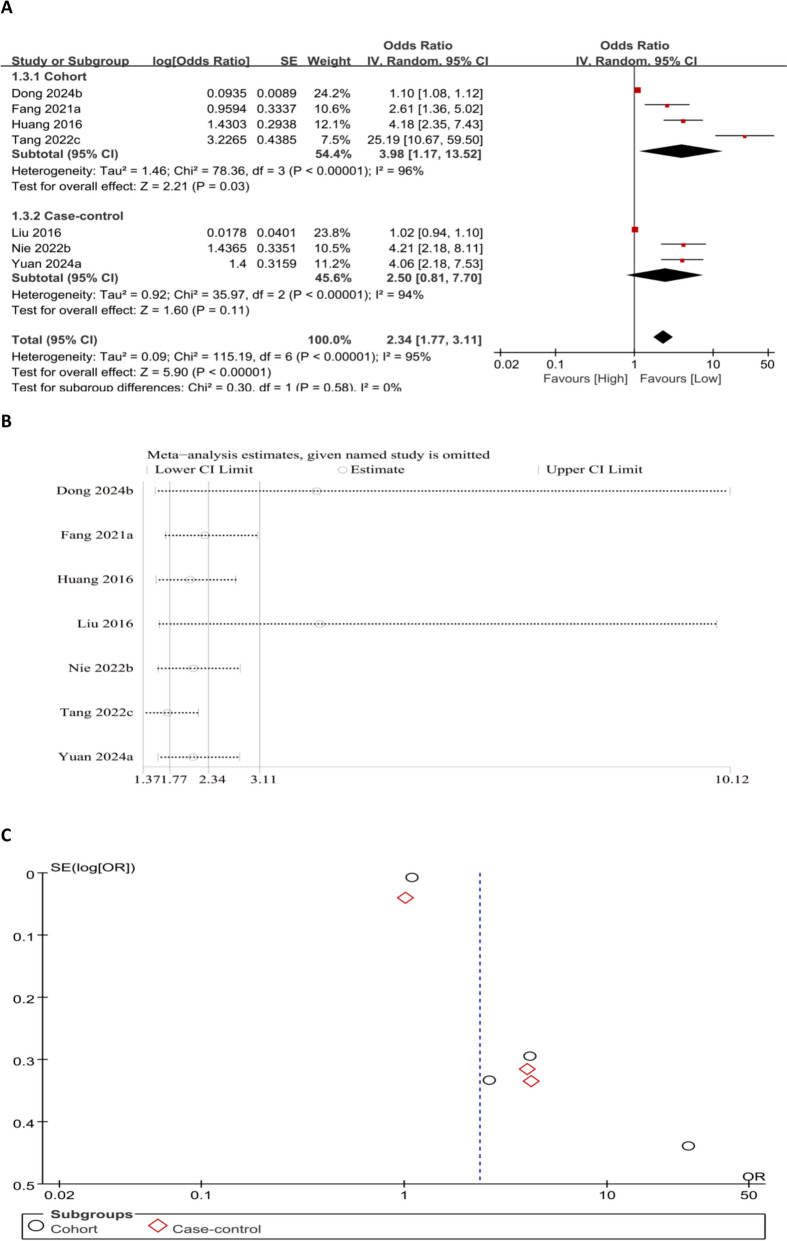
Forest plot (**A**), sensitivity analysis (**B**), and funnel plot (**C**) for NLR as a categorical variable, respectively

The meta-analysis using categorical variables demonstrated that elevated NLR levels were notably related to an increased OP risk (OR = 2.34, 95% CI 1.77–3.11;* P* < 0.00001) (Fig. 2A). The robustness of our results was rated via sensitivity analyses. After every study was sequentially omitted, the pooled effect estimates remained stable within the original range, indicating that no single study unduly influenced the overall association between NLR and OP risk. Although two studies [14, 32] contributed to substantial heterogeneity, the association between elevated NLR and increased OP risk remained both statistically significant and robust. Asymmetry in the funnel plot may suggest the presence of publication bias (Fig. 2B and C).

#### Association between NLR levels and OP risk (continuous variable)

**Fig. 3:**
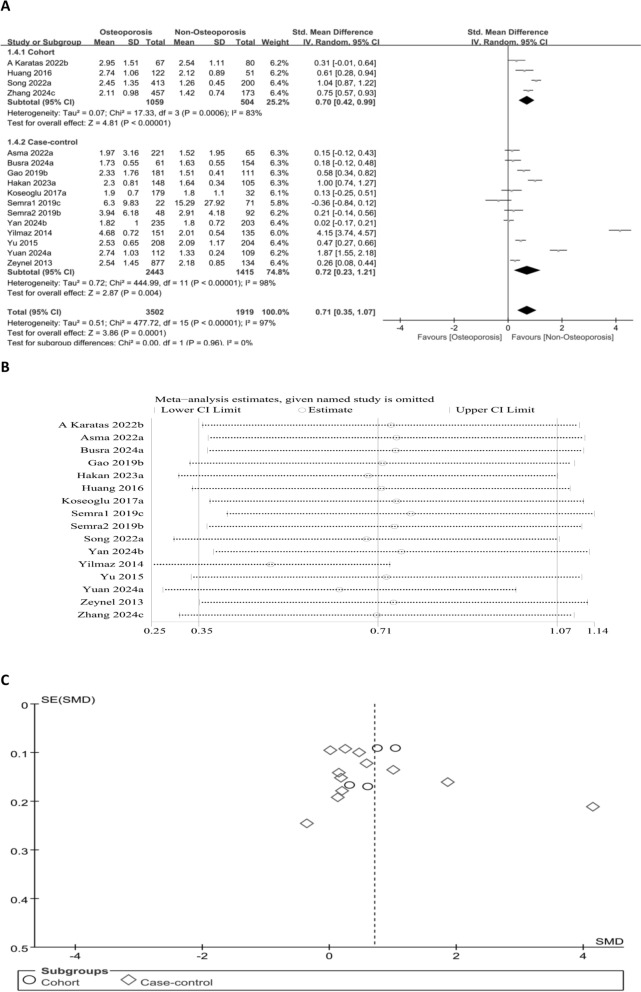
Forest plot (**A**), sensitivity analysis (**B**), and funnel plot (**C**) for NLR as a continuous variable, respectively

A forest plot displayed the relation of NLR to OP risk. OP sufferers exhibited markedly higher NLR levels than people without OP (SMD = 0.71, 95% CI 0.35–1.07; *P* = 0.0001) (Fig. 3A). This finding proves a significant positive link between elevated NLR levels and increased OP risk. To ensure the robustness of the results, sensitivity analyses were executed by removing individual studies one at a time. The pooled effect size remained consistent across iterations. Moreover, publication bias was detected via funnel plot analysis and Egger’s test, and significant publication bias was absent (Egger’s test: *P* = 0.448) (Fig. 3B and C).

#### Association between PLR and OP risk (categorical variable)

**Fig. 4:**
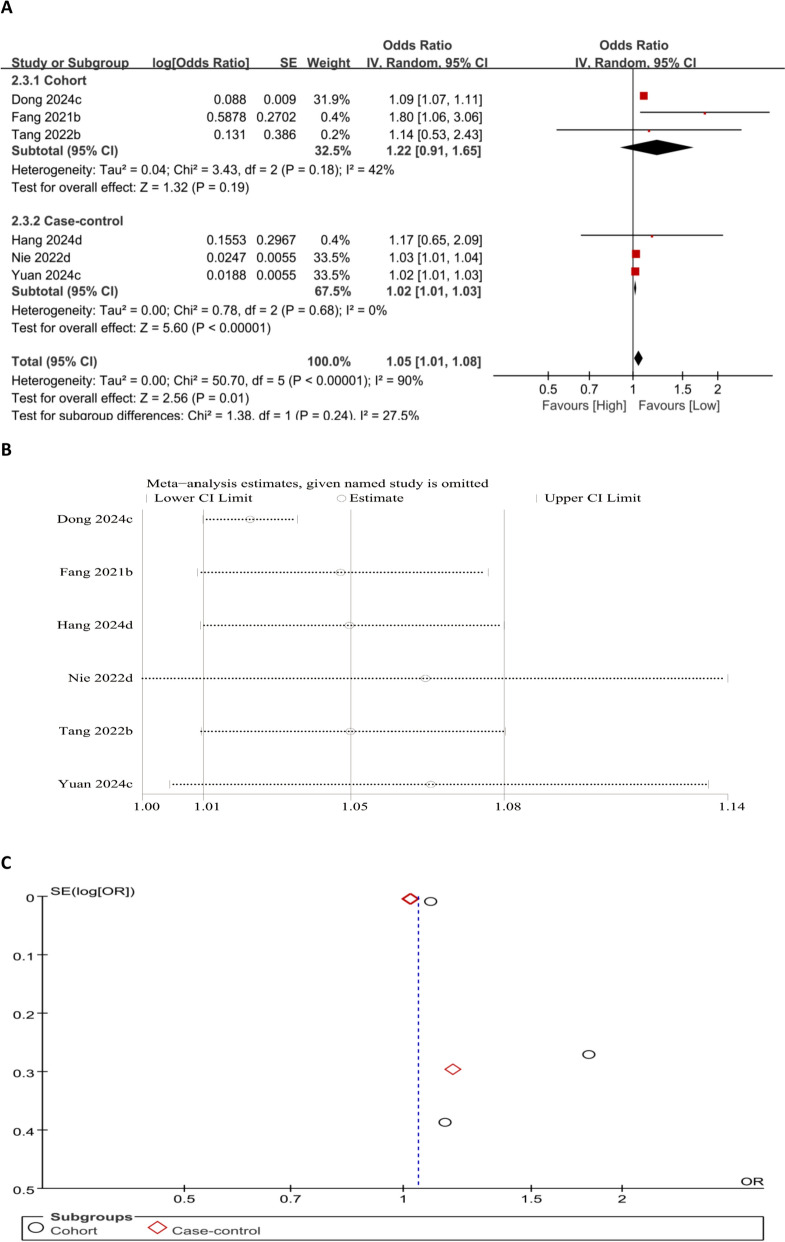
Forest plot (**A**), sensitivity analysis (**B**), and funnel plot (**C**) for PLR as a categorical variable, respectively

Our meta-analysis on PLR as a categorical variable suggests that PLR is a reliable indicator of OP risk. Elevated PLR levels markedly correlated with an elevated likelihood of OP (OR = 1.05, 95% CI 1.01–1.08; *P* = 0.01) (Fig. 4A). The foregoing findings indicate a positive relationship between increased PLR levels and OP development risk. To evaluate the robustness of this association, a sensitivity analysis was executed by sequentially removing individual studies and examining whether the pooled effect size remained within the original CI. The results revealed that after excluding the study by Nie [26], the effect size no longer remained consistent with the original range, so this study had a disproportionate influence on the pooled estimate. In the present study, the funnel plot appeared symmetrical, indicating a low likelihood of such bias (Fig. 4B and C).

#### Association between PLR level and OP risk (continuous variable)

**Fig. 5:**
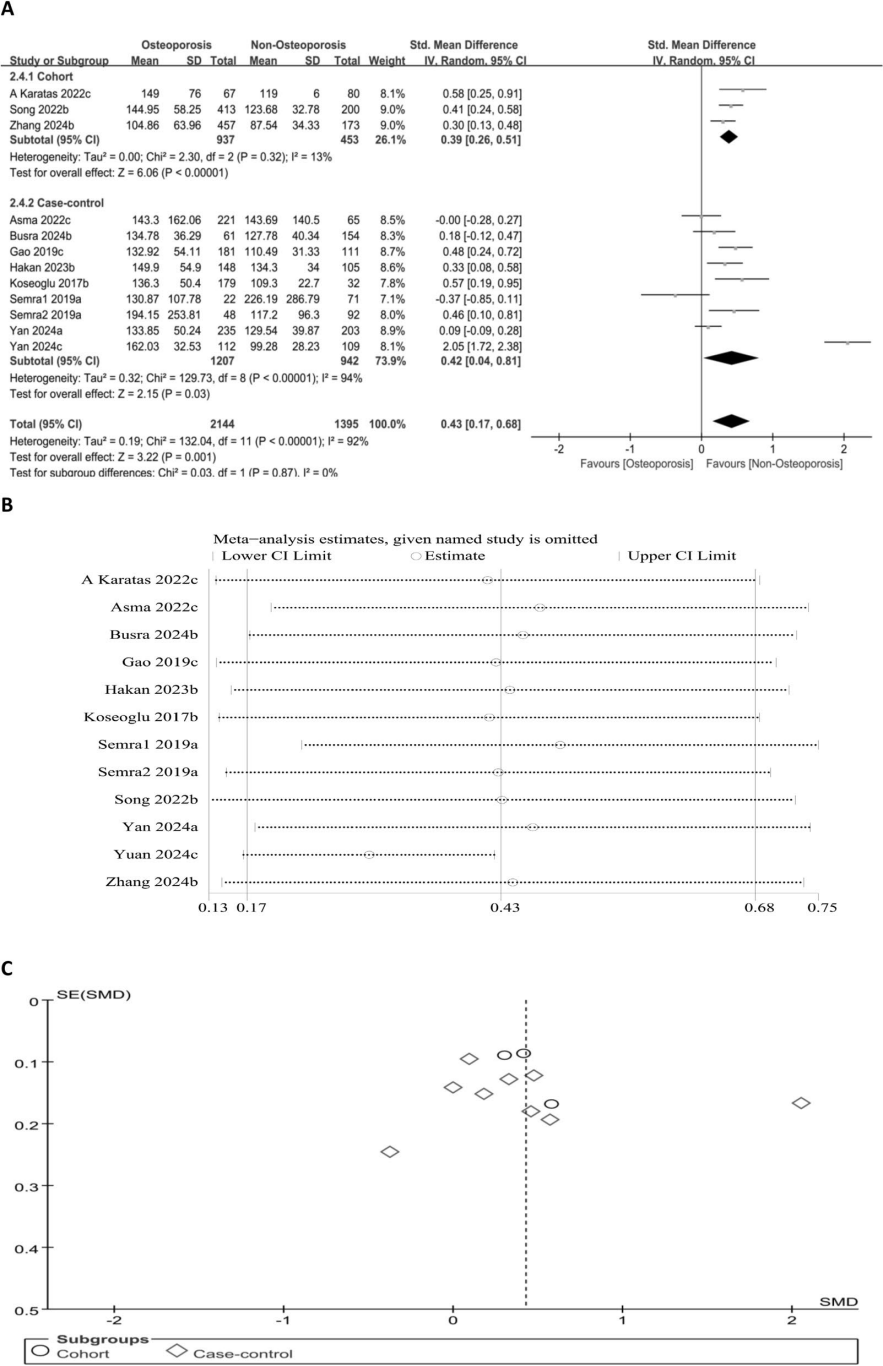
Forest plot (**A**), sensitivity analysis (**B**), and funnel plot (**C**) for PLR as a continuous variable, respectively

The relation of PLR to OP risk was examined via a forest plot. The OP cohort displayed notably higher PLR levels than the non-OP cohort (SMD = 0.43, 95% CI 0.17–0.68; *P* = 0.001) (Fig. 5A), which further verifies the significant link of elevated PLR to increased OP risk. Sensitivity analysis demonstrated that even when studies were removed one at a time, the association remained stable and statistically significant. Although the study by Yuan [22] contributed to substantial heterogeneity, the overall conclusion remained robust. Funnel plot symmetry and Egger’s test (*P* = 0.545) revealed no publication bias (Fig. 5B and C).

#### Association between MLR and the OP risk (categorical variable)

**Fig. 6:**
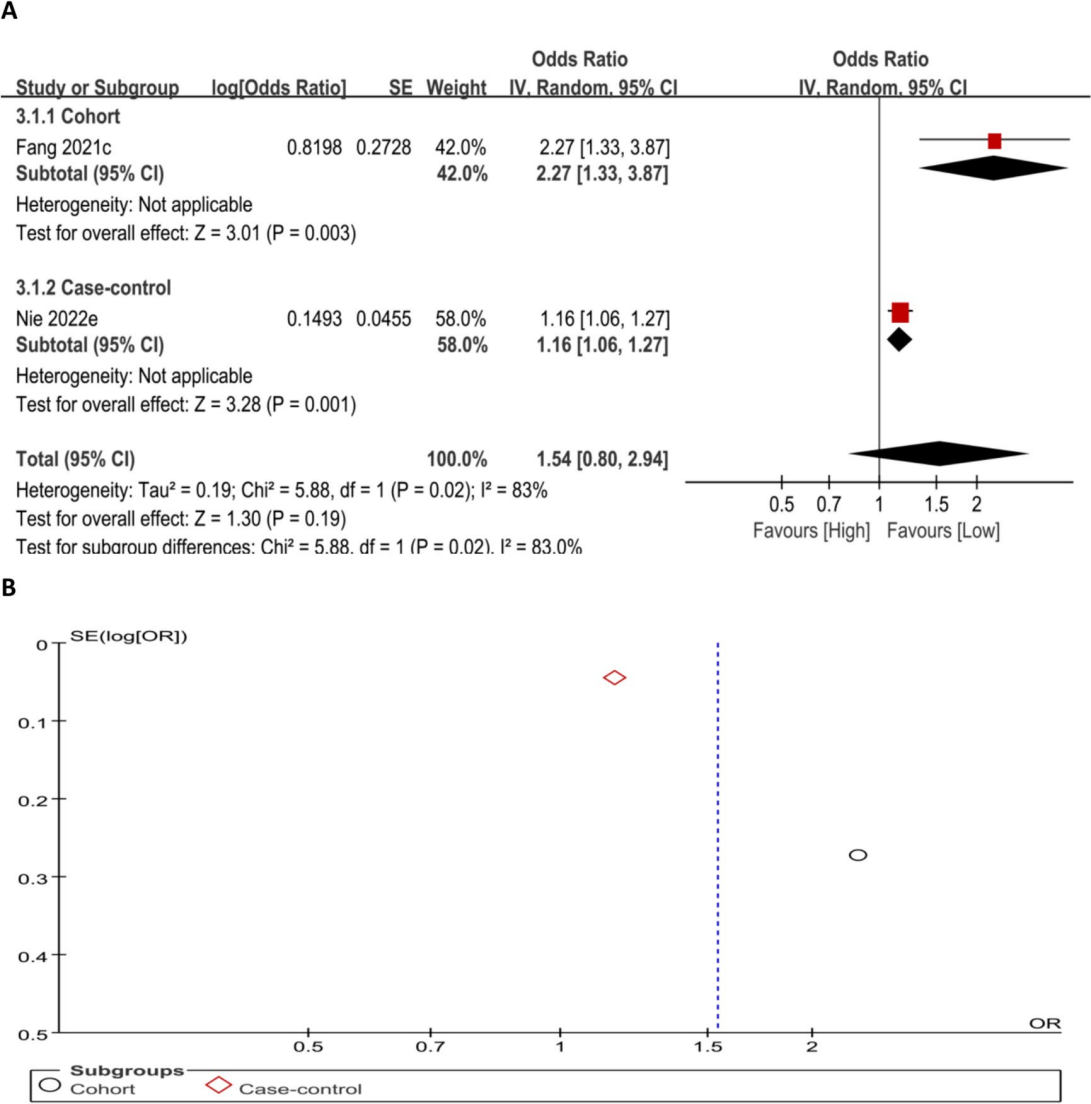
Forest plot (**A**) and funnel plot (**B**) for MLR as a categorical variable, respectively

The link of MLR as a categorical variable to OP risk was examined. The connection of higher MLR levels with the likelihood of OP was not statistically significant (OR = 1.54, 95% CI 0.80–2.94; *P* = 0.19) (Fig. 6A and B).

#### Association between MLR level and OP risk (continuous variable)

**Fig. 7:**
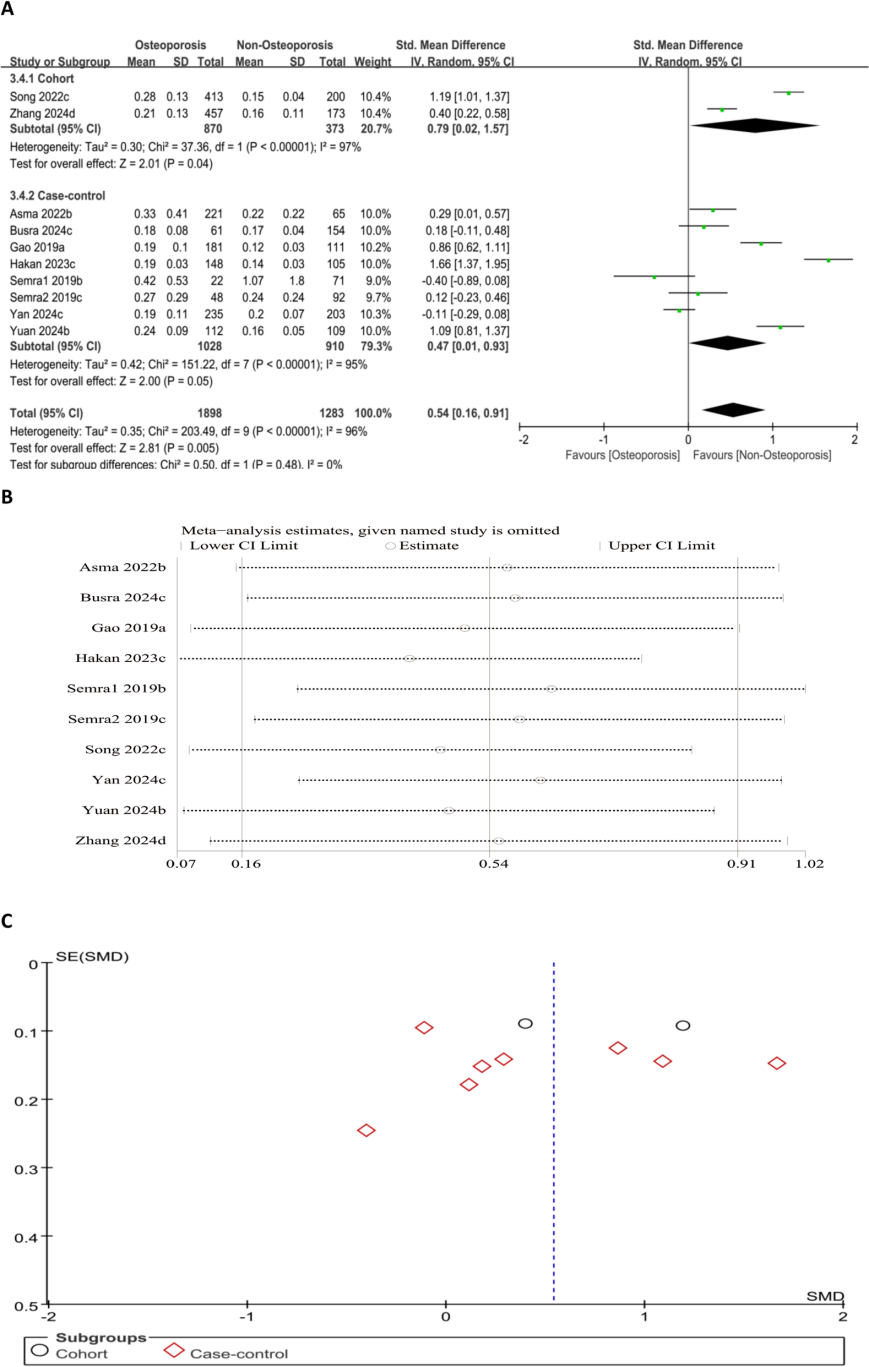
Forest plot (**A**), sensitivity analysis (**B**), and funnel plot (**C**) for MLR as a continuous variable, respectively

Forest plot analysis showed notably higher MLR levels in the OP sufferers than in the non-OP cohort (SMD = 0.54, 95% CI 0.16–0.91; *P* = 0.005) (Fig. 7A). This indicates a positive association between elevated MLR levels and OP risk. Sensitivity analysis confirmed that the effect sizes remained consistent when individual studies were removed one by one. Funnel plot symmetry and Egger’s test (*P* = 0.800) showed no notable publication bias (Fig. 7B and C).

#### Association between SII and the OP risk (categorical variable)

**Fig. 8:**
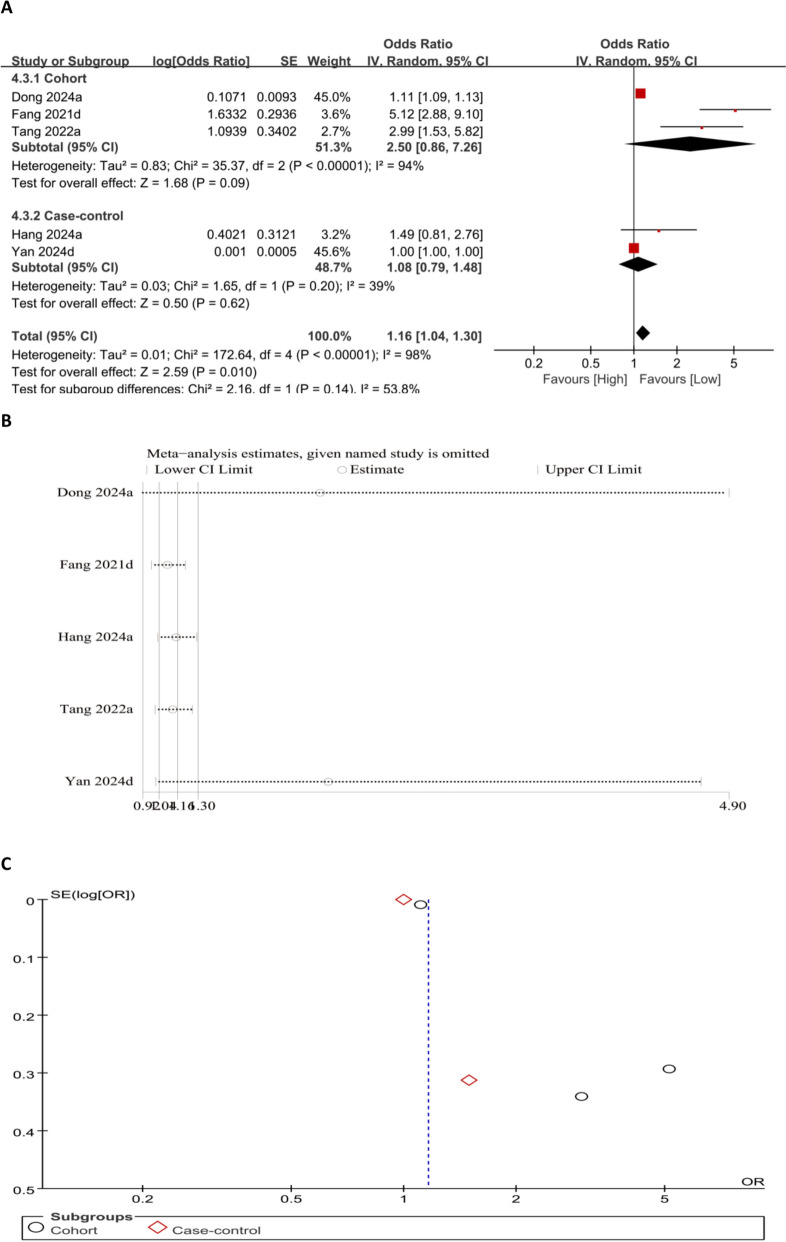
Forest plot (**A**), sensitivity analysis (**B**), and funnel plot (**C**) for SII as a categorical variable, respectively

Meta-analysis results of SII as a categorical variable showed that higher SII levels markedly correlated with elevated OP risk (OR = 1.16, 95% CI 1.04–1.30; *P* = 0.01) (Fig. 8A). Sensitivity analysis revealed that the exclusion of Di et al. [14] and Fang et al. [18] altered the pooled effect size beyond the original range, suggesting that these two studies exerted a disproportionate influence on the overall results. A symmetrical funnel plot indicates no publication bias (Fig. 8B and C).

#### Association between SII level and OP risk (continuous variable)

**Fig. 9:**
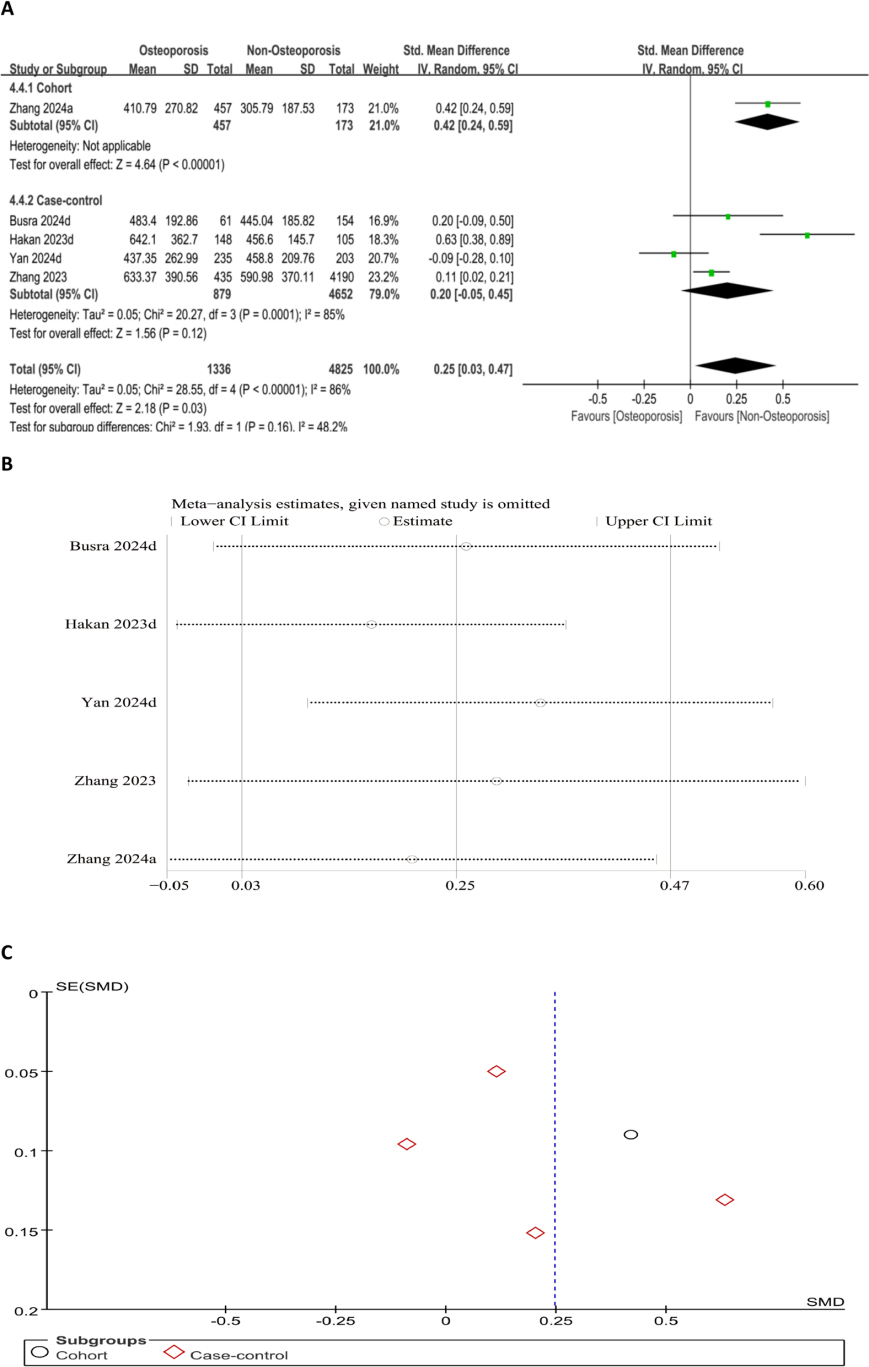
Forest plot (**A**), sensitivity analysis (**B**), and funnel plot (**C**) for SII as a continuous variable, respectively

The forest plot assessing the relationship between SII levels and OP risk demonstrated markedly higher SII levels in OP sufferers than non-OP individuals (SMD = 0.25, 95% CI 0.03–0.47; *P* = 0.03) (Fig. 9A). Sensitivity analysis revealed that excluding studies by Zhang et al. [12], Demir Cendek et al. [23], Zhang and Ni [24], and Yolaçan and Guler [25] affected the consistency of the pooled effect size, indicating these studies had a disproportionate influence. A symmetrical funnel plot indicates no publication bias (Fig. 9B and C).

#### Associations of SIRI and PIV with OP risk

**Fig. 10:**
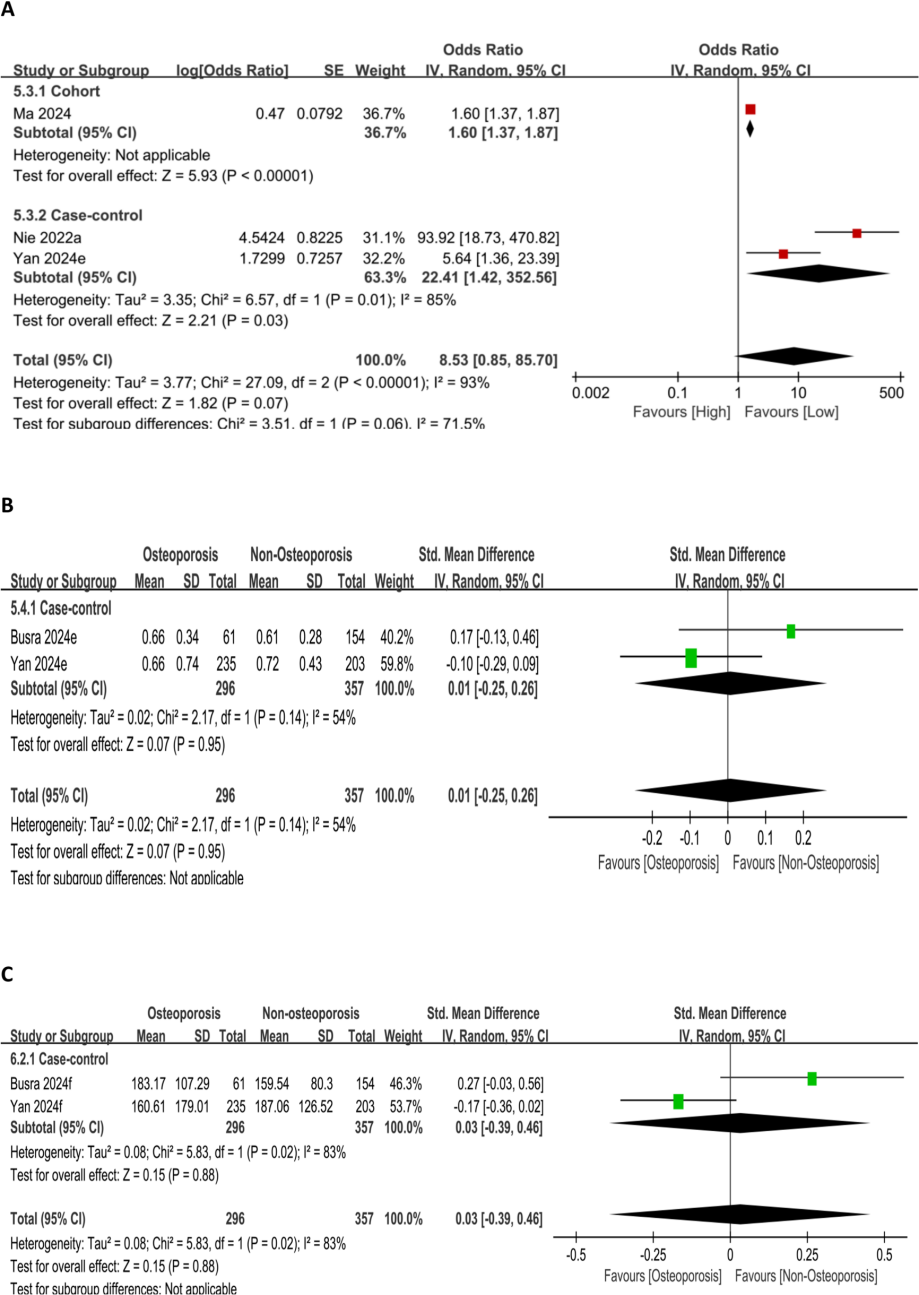
Forest plot for SIRI as a categorical variable (**A**) and SIRI (**B**) and PIV (**C**) as continuous variables, respectively

Analysis of SIRI as a categorical variable showed no statistically significant association with OP risk at elevated SIRI levels (*P* = 0.07) (Fig. 10A). Similarly, forest plot analysis exhibited no marked association between SIRI levels and OP risk (*P* = 0.95) (Fig. 10B), nor between PIV levels and OP risk (*P* = 0.88) (Fig. 10C).

### Subgroup analysis

Subgroup analyses were performed on continuous variables of NLR, PLR, and MLR to evaluate their predictive value under various conditions, including study type (cohort vs. case–control), sample size (≥ 400 vs. < 400), age (≥ 60 vs. < 60), geographical region (East Asia vs. West Asia), and specific populations (postmenopausal women, individuals with comorbidities). The prediction value of NLR for OP did not exhibit statistical significance among West Asians (*P* = 0.05) but remained significant across all other subgroups. PLR demonstrated significant predictive value for OP across all subgroups (*P* < 0.05). In contrast, MLR did not show significant predictive value in West Asia (*P* = 0.27) and < 60 age (*P* = 0.19) subgroups, while being predictive in all other subgroup analyses (Table 2).

**Table 2:** Subgroup analysis table for NLR, PLR, and MLR continuous variables

Subgroup	NLR (continuity variable)				PLR (continuity variable)				MLR (continuity variable)			
	Study group	SMD [95%CI]	*P* value (*Z*-test)	*I*^*2*^	Study group	SMD [95%CI]	*P* value (*Z*-test)	*I*^*2*^	Study group	SMD [95%CI]	*P* value (*Z*-test)	*I*^*2*^
Total	16	0.71 [0.35, 1.07]	< 0.00001	97%	12	0.43 [0.17, 0.68]	0.001	92%	10	0.91 [0.41–2.02]	0.005	96%
Study design												
Cohort	4	0.70 [0.42, 0.99]	< 0.00001	83%	3	0.39 [0.26, 0.51]	< 0.00001	13%	2	0.79 [0.02, 1.57]	0.04	97%
Case–control	12	0.72 [0.23, 1.21]	0.004	98%	9	0.42 [0.04, 0.81]	0.03	94%	8	0.47 [0.01, 0.93]	0.05	95%
Sample size												
≥ 400	8	0.96 [0.41, 1.52]	0.0007	98%	5	0.24[0.09, 0.39]	0.001	60%	5	0.68 [0.09, 1.28]	0.02	97%
< 400	8	0.45 [0.02, 0.88]	0.04	93%	7	0.57 [0.06, 1.08]	0.03	94%	5	0.39 [− 0.09, 0.88]	0.11	91%
Mean/median age												
≥ 60 year	8	1.11 [0.51, 1.71]	0.0003	98%	5	0.66[0.12, 1.20]	0.02	96%	4	0.74 [0.28, 1.21]	0.002	94%
< 60 year	8	0.31 [0.03, 0.60]	0.03	87%	7	0.27[0.08, 0.45]	0.005	65%	6	0.39 [− 0.20, 0.98]	0.19	96%
Region												
East Asia	7	0.75 [0.38, 1.13]	< 0.0001	95%	5	0.65 [0.17, 1.14]	0.008	96%	5	0.68 [0.18, 1.18]	0.007	97%
West Asia	9	0.67 [− 0.00, 1.34]	0.05	98%	7	0.26 [0.05, 0.48]	0.02	66%	5	0.38 [− 0.30, 1.06]	0.27	95%
Population												
Postmenopausal women	9	0.84 [0.26, 1.42]	0.004	98%	6	0.28 [0.14, 0.42]	< 0.00001	36%	5	0.53 [0.02, 1.05]	0.04	95%

*SMD* standardized mean differences, *95%CI* corresponding 95% confidence intervals, *NLR* neutrophil-to-lymphocyte ratio, *PLR* platelet-to-lymphocyte ratio, *MLR* monocyte-to-lymphocyte ratio

## Discussion

Our meta-analysis systematically proved the strong relations of elevated NLR, PLR, MLR, and SII to an increased OP risk. These findings offer novel insights into the role of various immune-inflammatory markers in OP pathogenesis and may inform future diagnostic and therapeutic strategies. NLR, a simple and cost-effective marker of systemic inflammation, is extensively employed in prognostic assessments of malignancy-related diseases [36]. NLR may reflect the complex interplay between the immune system and bone metabolism [37], potentially mediated by pro-resorptive cytokines that are elevated during inflammation. Our analysis revealed an evident link between higher NLR levels and the elevated likelihood of OP, a finding consistent with previous studies [8], thereby supporting the prospective utility of NLR as a robust indicator for OP risk assessment. Moreover, sensitivity analyses and publication bias assessments indicated that despite the presence of some publication bias, the association between NLR and OP remained stable and reliable.

Emerging evidence suggests that platelets, beyond their classical role in hemostasis, may also contribute to bone metabolism by releasing various growth factors and cytokines [38]. Chen [6] in a cohort study involving 9054 participants, proposed PLR as a prospective inflammatory predictor for OP. While the role of PLR as a surrogate marker of inflammation and immune status in OP remains to be fully elucidated, our pooled analysis demonstrated a significant link of raised PLR levels to elevated OP risk. Publication bias assessments supported the reliability of the overall findings, though the influence of certain individual studies could not be overlooked. Sensitivity analyses further highlighted the impact of specific studies on statistical significance, suggesting that the association between PLR and OP risk should be interpreted with caution. Although the overall correlation was statistically significant, its robustness is questionable due to the disproportionate influence of key studies. Therefore, a definitive causal relationship between PLR levels and OP risk cannot be firmly established at this stage. Future research should aim to address the potential sources of this instability, for example, by incorporating studies with diverse characteristics to enhance generalizability or by conducting more refined subgroup and meta-regression analyses to identify and account for heterogeneity. Additionally, alternative statistical approaches should be considered to adjust for influential studies and enable a more accurate evaluation of the PLR–OP relationship.

As a composite indicator of immune-inflammatory status, SII [39] is a prognostic marker in varied malignancies and other diseases [40]. Elevated SII levels may reflect both heightened systemic inflammation and a state of immune suppression. In the context of OP, SII may capture broader aspects of immune-inflammatory dysregulation. Our findings identified a significant relation of higher SII levels to elevated OP risk. While publication bias assessments suggested the overall reliability of this result, sensitivity analyses again indicated that certain studies exerted substantial influence on the pooled estimates. SIRI and PIV are relatively novel markers, with limited research available concerning their roles in OP. In our analysis, no marked relation was found between SIRI and OP risk, which may be attributable to the nature of its components or its yet-to-be-clarified mechanistic role in bone metabolism. Notably, although PIV has been recognized as a risk factor in other diseases [41], Yan [21], in a retrospective study, evaluated PIV’s predictive value in OP using area under the curve (AUC) analysis. Their findings suggest that when combined with age, PIV possesses notable diagnostic potential for abnormal BMD and may serve as a protective factor against OP. However, this observation lacks broader empirical support and requires validation through future investigations to further elucidate the role and underlying mechanisms of PIV in OP.

Research on MLR in OP remains scarce. Our meta-analysis is among the first to report a notable connection between elevated MLR levels and increased OP risk. This novel finding possibly presents fresh insights into the immune-inflammatory mechanisms underlying OP. However, although our sensitivity analyses and assessments of publication bias indicated a generally robust association, the influence of individual studies must still be considered when the foregoing results are interpreted.

Subgroup analyses further elucidated the influence of study design, sample size, age, geographic region, and specific populations on the relations of immune-inflammatory indices to the likelihood of OP. In cohort studies, heterogeneity was relatively low (*I*2 = 13%), suggesting that these inflammatory markers demonstrate more consistent predictive value within this study design. Cohort studies are generally regarded as higher-quality evidence due to their ability to capture temporal trends in biomarker fluctuations through prolonged follow-up periods. Notably, heterogeneity was also lower among postmenopausal women (*I*2 = 36%), a population at high risk for OP. Hormonal changes following menopause are closely linked to alterations in both BMD and inflammatory markers [42]. Qu [43] in a follow-up of 2834 women, noted the strong connections of NLR, MLR, SIRI, and AISI with all-cause mortality among postmenopausal females suffering from OP or osteopenia (*P* < 0.05). These findings suggest that, within this specific population, future research should further investigate the potential mechanisms underlying heterogeneity to enhance the predictive accuracy of inflammatory markers across different populations and inform the development of personalized therapeutic strategies. It has been shown that genetic variation may influence levels of inflammatory markers. A genetic study involving multi-ethnic populations reported that gene polymorphisms may be related to NLR levels [44]. Additionally, environmental and lifestyle factors could modulate the relation of NLR to OP. The lack of statistical significance for the prediction values of NLR and MLR within the West Asian population may reflect a complex interplay of multiple factors, including genetic background, environmental exposures, lifestyle habits, and disparities in healthcare resources. These findings highlight the need to consider regional specificity and individual differences when applying such biomarkers to more accurately assess disease risk and prognosis.

In recent years, more focus has been placed on the link between inflammation and bone loss in the field of OP research. Specifically, immune-inflammatory markers have garnered growing interest. In the meta-analysis by Liu [7], all ten included studies were case–control in design and involved only 2616 participants. Similarly, the meta-analysis by Salimi et al. [8] incorporated eight studies: four case–control and four cohort studies. Our meta-analysis encompassed 24 studies, among which 13 focused on postmenopausal women (eight case–control and five cohort studies). Our findings further verify the links of NLR and PLR to OP and extend the current understanding of newer immune-inflammatory indices. Building upon literature published before 2022 and incorporating recent advancements, our study evaluated not only NLR and PLR but also a broader range of inflammatory markers like MLR, SII, SIRI, and PIV. These indices, as emerging areas of investigation, are gaining attention for their potential relevance in OP. They are believed to play a critical role in immune regulation among OP patients, reflecting the systemic inflammatory status through the balance between pro- and anti-inflammatory responses. As novel indicators for assessing immune response and inflammation, they are increasingly being incorporated into OP-related research. Their significantly elevated levels in OP patients suggest that these indices possibly have an active role in the immunopathogenesis of OP, though the relationship is complex and multidimensional.

Despite the inclusion of various immune-inflammatory indices and their associations with OP risk, several limitations exist. First, the limited number of available studies possibly result in insufficient statistical power for certain indices. Second, the predominance of Asians in eligible studies may limit the generalizability. Moreover, owing to data constraints, it was impossible to assess the impact of race, lifestyle, and genetic factors on the connections of inflammatory indices with OP risk. Additionally, since our meta-analysis utilized published literature, certain publication bias cannot be ruled out. Future research should seek to elucidate the biological mechanisms underlying the links of immune-inflammatory indices to OP and to validate these findings in large-scale, multi-ethnic, multicenter cohort studies. Furthermore, investigations into the effects of lifestyle interventions on immune-inflammatory status and their long-term implications for OP risk are warranted.

## Conclusion

This meta-analysis proves that NLR, PLR, MLR, and SII are significantly linked to OP risk, whereas SIRI and PIV show no clear relationship. Subgroup analyses indicate that study design and geographic region may be key factors influencing these associations. Given the potential heterogeneity, publication bias, and retrospective nature of the included studies, further international, multicenter, prospective clinical research is necessary to verify the relations of immune-inflammatory indices to OP risk. Future efforts should focus on determining the optimal cut-off values and applicable populations for predictive models to identify high-risk individuals early and offer targeted interventions, ultimately easing the burden of OP.

Future studies should further explore the underlying biological mechanisms linking immune-inflammatory indices and OP risk. Large-scale, multi-center, and multi-ethnic cohort studies are warranted to validate the present findings. Furthermore, to precisely evaluate OP risk, comprehensive clinical datasets incorporating NLR, PLR, MLR, SII, and corresponding BMD measurements should be collected. Receiver operating characteristic (ROC) curve analysis should be employed to evaluate the trade-off between sensitivity and specificity across varying thresholds, thereby identifying optimal cut-off values. In addition, constructing cytokine network models to elucidate interrelationships among cytokines and immune-inflammatory indices will aid in delineating relevant inflammatory signaling pathways. Integrating statistical analysis with mechanistic insights will ultimately enhance the scientific rigor and precision of OP assessment and therapeutic strategies.

## Supplementary Information

Supplementary Material 1

Supplementary Material 2

Supplementary Material 3

Supplementary Material 4

Supplementary Material 5

## Data Availability

All data analyzed in this meta-analysis were extracted from previously published studies cited in the reference list; the extracted dataset is provided in the Supplementary Material.

## Abbreviations

OP: Osteoporosis
NLR: Neutrophil-to-lymphocyte ratio
PLR: Platelet-to-lymphocyte ratio
MLR: Monocyte-to-lymphocyte ratio
SII: Systemic immune-inflammation index
SIRI: Systemic inflammatory response index
PIV: Pan-immune-inflammation value
CBC: Complete blood count
PRISMA: Preferred Reporting Items for Systematic Reviews and Meta-Analyses
WHO: World Health Organization
DXA: Dual-energy X-ray absorptiometry
CI: Confidence interval
SMD: Standardized mean difference
NOS: Newcastle–Ottawa Scale
ROC: Receiver operating characteristic
