# Supplementary material for: Association between immune-inflammatory index and osteoporosis: a systematic review and meta-analysis
Source: Eur J Med Res. 2025 Jul 16;30:632. doi: 10.1186/s40001-025-02893-w (PMC12265382; doi:10.1186/s40001-025-02893-w)
Supplement: Supplementary file 1 — Supplementary Material 1 [file 40001_2025_2893_MOESM1_ESM.docx]

Supplementary Table S1.the literature search strategy

Pubmed-138

((("Lymphocytes"[Mesh]) OR (((Lymphocyte) OR (Lymphoid Cells)) OR (Lymphoid Cell))) AND (Ratio)) AND (("Osteoporosis"[Mesh]) OR (((((((((Age-Related Osteoporosis) OR (Age-Related Osteoporoses)) OR (Age Related Osteoporosis)) OR (Age-Related Bone Loss)) OR (Age-Related Bone Losses)) OR (Senile Osteoporoses)) OR (Senile Osteoporosis)) OR (Post-Traumatic Osteoporoses)) OR (Post-Traumatic Osteoporosis)))

Embase-387

(((Lymphocytes) OR (((Lymphocyte) OR (Lymphoid Cells)) OR (Lymphoid Cell))) AND (Ratio)) AND ((Osteoporosis) OR (((((((((Age-Related Osteoporosis) OR (Age-Related Osteoporoses)) OR (Age Related Osteoporosis)) OR (Age-Related Bone Loss)) OR (Age-Related Bone Losses)) OR (Senile Osteoporoses)) OR (Senile Osteoporosis)) OR (Post-Traumatic Osteoporoses)) OR (Post-Traumatic Osteoporosis)))


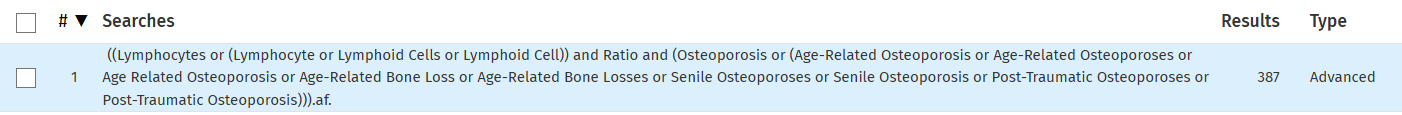


Cochrane-9

(((Lymphocytes) OR (((Lymphocyte) OR (Lymphoid Cells)) OR (Lymphoid Cell))) AND (Ratio)) AND ((Osteoporosis) OR (((((((((Age-Related Osteoporosis) OR (Age-Related Osteoporoses)) OR (Age Related Osteoporosis)) OR (Age-Related Bone Loss)) OR (Age-Related Bone Losses)) OR (Senile Osteoporoses)) OR (Senile Osteoporosis)) OR (Post-Traumatic Osteoporoses)) OR (Post-Traumatic Osteoporosis)))


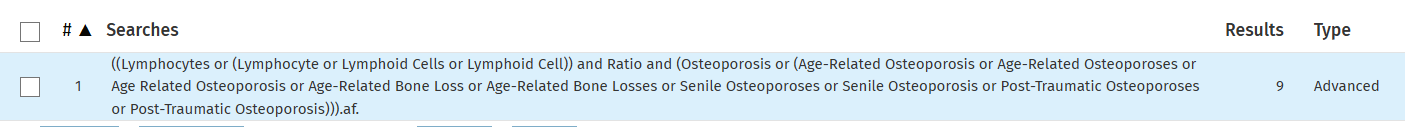


Web of Science-96

(((Lymphocytes) OR (((Lymphocyte) OR (Lymphoid Cells)) OR (Lymphoid Cell))) AND (Ratio)) AND ((Osteoporosis) OR (((((((((Age-Related Osteoporosis) OR (Age-Related Osteoporoses)) OR (Age Related Osteoporosis)) OR (Age-Related Bone Loss)) OR (Age-Related Bone Losses)) OR (Senile Osteoporoses)) OR (Senile Osteoporosis)) OR (Post-Traumatic Osteoporoses)) OR (Post-Traumatic Osteoporosis))) (Topic)
