# Supplementary material for: Association between immune-inflammatory index and osteoporosis: a systematic review and meta-analysis
Source: Eur J Med Res. 2025 Jul 16;30:632. doi: 10.1186/s40001-025-02893-w (PMC12265382; doi:10.1186/s40001-025-02893-w)
Supplement: Supplementary file 2 — Supplementary Material 2 [file 40001_2025_2893_MOESM2_ESM.docx]

| Supplementary Table S2. Quality evaluation of the eligible Cohort with Newcastle–Ottawa scale. | | | | | | | | | |
| --- | --- | --- | --- | --- | --- | --- | --- | --- | --- |
| Study | Selection | | | | Comparability | | Outcome | | |
|  | Representative-ness | Selection of  non-exposed | Ascertainment  of exposure | Outcome not present at start | Comparability on most important factors | Comparability on other risk factors | Assessment of outcome | Long enough follow-up (median≥1 year) | Adequacy  (completeness) of follow-up |
| Zhang 2024[12] | * | * | * | * | - | * | * | * | * |
| Ma 2024[13] | * | * | * | * | * | * | * | * | * |
| Dong 2024[14] | * | * | * | * | - | - | * | * | * |
| Tang 2022[15] | * | * | * | * | * | - | * | - | * |
| Song 2022[16] | * | - | * | * | * | - | * | * | * |
| A Karatas 2022[17] | * | * | * | * | - | * | * | - | * |
| Fang 2021[18] | * | * | * | * | * | - | * | * | * |
| Huang 2016[19] | * | * | * | * | - | - | * | * | * |
| *indicates criterion met; - indicates significant of criterion not met. | | | | | | | | | |
