# Supplementary material for: Association between immune-inflammatory index and osteoporosis: a systematic review and meta-analysis
Source: Eur J Med Res. 2025 Jul 16;30:632. doi: 10.1186/s40001-025-02893-w (PMC12265382; doi:10.1186/s40001-025-02893-w)
Supplement: Supplementary file 3 — Supplementary Material 3 [file 40001_2025_2893_MOESM3_ESM.docx]

| Supplementary Table S3. Quality evaluation of the eligible Case-control with Newcastle–Ottawa scale. | | | | | | | | | |
| --- | --- | --- | --- | --- | --- | --- | --- | --- | --- |
| Study | Selection | | | | Comparability | | Exposure factor Outcome | | |
|  | Case confirmation | Representativeness of cases | Control group selection | Determination of control group | Comparability on most important factors | Comparability on other risk factors | Identification of exposure factors | Use the same method to determine | Non-response rate |
| Hang 2024[20] | * | * | * | * | - | - | * | * | * |
| Yan 2024[21] | * | * | * | * | - | - | * | * | * |
| Yuan 2024[22] | * | * | * | * | - | - | * | * | * |
| Busra 2024[23] | * | * | * | * | - | - | * | * | * |
| Zhang 2023[24] | * | * | * | * | * | - | * | * | * |
| Hakan 2023[25] | * | * | * | * | * | - | * | * | * |
| Nie 2022[26] | * | * | * | * | - | - | * | * | * |
| Asma 2022[27] | * | * | * | * | - | - | * | * | * |
| Gao 2019[28] | * | * | * | * | - | - | * | * | * |
| Semra1 2019[29] | * | * | * | * | - | - | * | * | * |
| Semra2 2019[30] | * | * | * | * | - | - | * | * | * |
| Koseoglu 2017[31] | * | * | * | * | * | - | * | * | * |
| Liu 2016[32] | * | * | * | * | - | - | * | * | * |
| Yu 2015[33] | * | * | * | * | - | - | * | * | * |
| Yilmaz 2014[34] | * | * | * | * | - | - | * | * | * |
| Zeynel 2013[35] | * | * | * | * | - | - | * | * | * |
| *indicates criterion met; - indicates significant of criterion not met. | | | | | | | | | |
