# Supplementary material for: Association between immune-inflammatory index and osteoporosis: a systematic review and meta-analysis
Source: Eur J Med Res. 2025 Jul 16;30:632. doi: 10.1186/s40001-025-02893-w (PMC12265382; doi:10.1186/s40001-025-02893-w)
Supplement: Supplementary file 4 — Supplementary Material 4 [file 40001_2025_2893_MOESM4_ESM.docx]

**Supplementary Table S4.** Inclusion criteria and exclusion criteria for all literatures

| **Author** | **Inclusion criteria** | **Exclusion criteria** |
| --- | --- | --- |
| Zhang 2024[12] | This retrospective cohort study was conducted among postmenopausal women who underwent DXA scanning at the Third Hospital of Hebei Medical University, a general hospital with specialized orthopedics and endocrinology services. | Women with a history of menopause < 1 year; factors or conditions that affect the immunoinflammatory response, including hepatic, renal, hematological, oncological, and rheumatologic diseases; history of steroid use, trauma, or blood transfusions over the last 12 months; and use of anti-osteoporotic treatment within six months before inclusion. |
| Ma 2024[13] | Elderly hypertensive patients (age ≥ 60 years) who attended hospital between January 2021 and December 2023 and completed BMD screening were included. | Miss information of lymphocyte count,white blood cell count and monocytes count. |
| Dong 2024[14] | The UK Biobank gathered detailed information on various phenotypes and genotypes from nearly 500000 individuals at 22 assessment centres across the United Kingdom. All participants provided written informed consent. | First, author excluded 10634 participants with OP at baseline. Next, author excluded subjects without systemic inflammation markers and physical frailty data, creating systemic inflammation (N = 393443) and frailty (N = 390485) subcohorts. From these subcohorts, author further excluded those with a fracture history at baseline to explore the association of systemic inflammation, frailty and fractures. |
| Tang 2022[15] | (1) postmenopausal women aged ≥ 50 years and (2) participants with complete BMD and SII data. | (1) participants who were pregnant, (2) participants who were diagnosed with RA by doctors, (3) participants who were diagnosed with cancer by doctors, (4) participants who had a history of female hormone use, and (5) participants who had a history of glucocorticoid use. |
| Song 2022[16] | Author investigated 413 consecutive postmenopausal patients with RA and 200 age-matched (±2 years) postmenopausal healthy controls who underwent DXA between January 2005 and December 2017. Postmenopausal status was defined as no menstruation for more than one year. All patients with RA were diagnosed by experienced rheumatologists at our hospital according to the 1987 American College of Rheumatology (formerly American Rheumatism Association) revised classification criteria for RA or the 2010 American College of Rheumatology/European League Against Rheumatism classification criteria.  Postmenopausal healthy controls who had undergone comprehensive routine health checkups at the health promotion center of the same hospital were randomly selected for this study. The control group had no history of rheumatic diseases, including RA, hematologic diseases, malignancies, active infection, or fragility fracture, and did not take anti-osteoporotic medications except for calcium and/or vitamin D, all of which could affect NLR, PLR, MLR, or BMD. | The following patients with RA were excluded from the analysis: (1) those with rheumatic diseases other than RA except for Sjogren’s syndrome; (2) those taking drugs for OP treatment including bisphosphonates, selective estrogen receptor modulators, denosumab, or teriparatide, except for calcium and/or vitamin D; (3) those with a history of spine and/or hip surgery or with metal implants in situ, which could affect the result of DXA examination; (4) those with a history of osteoporotic fractures at the spine or hip; (5) those with concomitant hematologic disorders, malignancies, or active infection; and (6) those with an eGFR less than 30 mL/min/1.73 m^2^ . |
| A Karatas 2022[17] | Author study was designed cross-sectional and conducted with 283 nondialysis CKD patients who applied to the Nephrology outpatient clinic between January 2019 and July 2019. None of the patients included in our study were using steroids. | Patients with serum PTH levels above 800 pg/ml were considered to have severe hyperparathyroidism and these patients were excluded from the study. Those with severe primary hyperparathyroidism, immobilization, Parkinson’s disease, multiple sclerosis, polio, amyotrophic lateral sclerosis, have a systemic disease were excluded from the study. Patients using steroids to ensure group homogenization were excluded from the study. The diagnosis of nondialysis CKD was made in accordance with the KDIGO guideline. The eGFR value of the patients was calculated. The eGFR value was calculated with Chronic Kidney Disease Epidemiology Collaboration creatinine equation. The eGFR value of all patients was <60 mL/min/1.73 m^2^ . The patients were Stage 3-5 patients according to the KDIGO guideline. Patients who received dialysis treatment were not included in the study. |
| Fang 2021[18] | The enrolled study participants were postmenopausal women older than 45 years old who had natural menopause for at least 1 year. | (a) participants who had endocrine or metabolic diseases, such as diabetes mellitus, thyroid or parathyroid diseases, and rheumatism; (b) participants who received calcium supplements or glucocorticoids; (c) participants who had clinical manifestations indicating recently acute or chronic infections; (d) participants who had solid or hematological malignancies; (e) participants who had obvious hepatorenal dysfunctions; and (f) participants who had incomplete information regarding clinical examinations. |
| Huang 2016[19] | Author screened two hundred and thirty three postmenopausal women at our medical centers in China, who were menopausal for at least three years and were eligible for enrollment if they were 45 to 79 years old, and categorized into osteoporosis group and control group (normal BMD and osteopenia), as BMD evaluation. None of those included were taking drugs or hormones that influence bone metabolism, such as glucocorticoids, estrogens, thyroid hormone, fluoride, bisphosphonate, calcitonin, thiazide diuretics, barbiturates, vitamin D, or calcium-containing drugs. | Those patients were excluded who had self-report diagnosis of T1D or T2D and/or they were taking antidiabetic medication and/or insulin or they had fasting plasma glucose concentration equal or more than 126 mg/dl. Those with clinical condition that affected bone metabolism, such as diseases of the liver, kidney, thyroid, or parathyroids, rheumatic diseases, malabsorption syndromes, malignant tumors, and hematological diseases were also excluded. |
| Hang 2024[20] | From January 2022 to May 2023, we selected 826 male patients aged 40 and above in the First Affiliated Hospital of Guangzhou University of Chinese Medicine. All participants enrolled in this study underwent the DXA scanning. | (1) patients who were younger than 40 years old; (2) patients who had the blood test within a week after surgery; (3) patients who are undergoing acute infections; and (4) patients who had missing data regarding clinical examinations. |
| Yan 2024[21] | 1. aged ≥ 50; (2) no history of diabetes; (3) complete clinical data. | (1) Presence of diseases such as infectious states or blood diseases; (2)Presence of other endocrine diseases affecting bone metabolism (thyroid diseases, parathyroid diseases, gonadal diseases); (3)Presence of tumor-related diseases such as multiple  myeloma; (4)History of radiotherapy or chemotherapy; (5) Presence of severe liver and kidney function abnormalities. |
| Yuan 2024[22] | The inclusion criteria were as follows: conformity with the T2DM diagnostic criteria recommended by the World Health Organization Diabetes Expert Committee in 1999; menopausal for more than 1 year; all patients have undergone comprehensive DXA screening; no missing clinical data. | Patients with other types of diabetes mellitus (DM) or acute complications of DM; presence of other endocrine system disorders; acute or chronic infectious diseases; hematological disorders; cancer; autoimmune diseases; severe hepatic or renal diseases; non-natural menopause; long-term use of medications affecting bone metabolism, such as corticosteroids, calcium supplements, vitamin D, sex hormones, and bisphosphonates. |
| Busra 2024[23] | This study included patients diagnosed with menopause who underwent DXA and blood tests to assess BMD and whose test results were available in the hospital’s electronic database. | Women were excluded if they had received hormone therapy during the reproductive period, were using corticosteroids, had a history of malignancy or fracture, experienced immobility (e.g. prolonged bed rest or wheelchair dependence), or had systemic diseases such as diabetes, hypertension, kidney disease, liver disease, or depression. Additionally, individuals with incomplete medical records, DXA, and laboratory results were also excluded. |
| Zhang 2023[24] | Patients over 45 years old with primary OP. | this study, and any factors related to secondary OP are excluded (including use of op and corticosteroid drugs, hyperparathyroidism, kidney disease, and so on). |
| Hakan 2023[25] | Patients who were registered in the archives of our hospital between January 1, 2021 and January 1, 2022, who had DXA examination on the same day and whose peripheral blood samples were taken, and who had a history of menopause for at least one year were included in the study. To obtain a comprehensive blood routine examination, venous blood samples of about 6 mL were collected from all participants after overnight fasting. | Patients who had menopause for less than one year, a history of acute or chronic infection, liver or kidney disease, rheumatological disease, autoimmune disease, cancer, hematological disease, steroid use, and blood transfusion within the last previous year. |
| Nie 2022[26] | (1) Age ≥ 60years)；(2) Participants were randomly recruited from among people receiving annual check-ups at the Physical Examination Center of The Second Affiliated Hospital of Harbin Medical University between January 2018 and December 2020. (3) DXA examination can be conducted and peripheral blood samples can be collected. | (1) refusal to give or legally incapable of giving informed consent; (2) body composition and bone density could not be accurately estimated due to lower limb injuries; (3) history of serious metabolic or endocrine diseases or fractures; and (4) recent or ongoing use of medications affecting bone metabolism, such as thiazolidinediones, glucocorticoids, statins, and antiepileptic medications. |
| Asma 2022[27] | A non-probability convenience sampling strategy was used to recruit all women presenting to this hospital during the above-mentioned period who were either ≥ 50 years of age or < 50 years of age if postmenopausal status was confirmed. | Women with a history of menopause of less than a year in duration were excluded, as were women with conditions or factors thought to affect immunoinflammatory response, including those with hepatic, renal, oncological, haematological or rheumatologic diseases. Similarly, women with a history of steroid use, trauma, hospitalisation over the preceding six months and blood transfusions over the last 12 months were also excluded. |
| Gao 2019[28] | Three hundred sixteen inpatients who were treated at the Department of Orthopedics, Shenzhen Hospital of Traditional Chinese Medicine between January 1, 2015 and August 31, 2018 were included in this cross-sectional study. All patients diagnosed with osteoporosis fulfilled the 2015 Guidelines for the Diagnosis and Treatment of Osteoporosis issued by the Branch of  OP and Bone Mineral Salt Diseases, Chinese Medical Association.  One hundred eleven healthy control subjects who were referred to our hospital for routine checkups with normal BMD levels were recruited. In this study, written or verbal consent was obtained from all the patients. | Those patients with hypertension (n=52), acute inflammatory disease (n=43), malignancy tumor (n=4), autoimmune disease (n=9), hematological disorders (n=6) (n=6), renal and liver failure (n=8), thyroid or parathyroid disorders (n=5), and infection (n=8) were excluded. |
| Semra1 2019[29] | The sample consisted of 93 females aged 18-40 years and in the first month of the postpartum period. All the women had low back pain. The BMD Z-score values of the lumbar vertebrae, femur (neck and total) were examined using dual energy x-ray absorbtiometry four weeks after birth. | The sample excluded all individuals currently using antiresorbtive drugs or taking vitamin D or calcıum medication or with acute or chronic infections, history of trauma or psychiatric disorders or any secondary causes of inflammation (e.g. chronic hepatic, renal or thyroid disease, smoking, diabetes or haematological disease and malignancies). |
| Semra2 2019[30] | All postmenopausal females, 252 outpatients who were admitted to the obstetrics and gynecology and physical therapy clinics between July 2016 and December 2017 were retrospectively analyzed. While the patients who were known to have been menopausal for at least one year were including. | The patients who have <1 year history of menopause, acute or chronic infection, anemia, thrombocytopenia and leucopenia, history of oncologic or hematologic diseases, liver or kidney disease, rheumatologic disease, any trauma history requiring hospitalization in the last 6 months, history of blood transfusion in the last one year and steroid use were excluded in the study. |
| Koseoglu 2017[31] | Electronic records between May 2015 and May 2016 were analyzed retrospectively. Informed consent was obtained from all participants involved. In total, 211 patients, aged between 50 and 63, were included in the study. The patients who have ＞1 year history of menopause. | The patients with secondary causes of inflammation such as hematologic or cardiovascular diseases, diabetes, kidney–liver diseases, asthma, obesity, smoking and neoplastic diseases and patients with hormone replacement treatment were excluded from the study. |
| Liu 2016[32] | Two-hundred sixty-nine postmenopausal women, aged 45–60 years, who were admitted to the outpatient clinic of gynecology department of Hangzhou Hospital of Nanjing Medical University, were enrolled in this study from January 2013 to December 2014. All of the subjects had experienced natural menopause; menopause was defined as 1 year with no  menstrual bleeding. | Exclusion criteria were alcohol consumption, tobacco use, taking drugs (especially drugs which affect bone density and calcium metabolism), application of MHT and other systemic diseases, such as diabetes, coronary heart disease, heart failure, tumor. |
| Yu 2015[33] | The study enrolled 512 postmenopausal women with similar education background and income from the International Physical Examination and Healthy Center of our hospital between Jan. 2009 to Dec. 2010. DXA examination can be conducted and peripheral blood samples can be collected. | Exclusion criteria include smoking, active inflammation, chronic inflammatory diseases, chronic liver and kidney diseases, hematological disorders, fractures, cancer, coronary atherosclerotic heart disease, stroke, peripheral arterial occlusion, and medical treatment with hormone-replacement therapy, glucocorticoid drugs and statins. |
| Yilmaz 2014[34] | The study enrolled 438 postmenopausal women with DXA examination can be conducted and peripheral blood samples can be collected. | Chronic obstructive pulmonary disease, hematological disorders, autoimmune diseases, valvular diseases, thyroid or parathyroid disorders, rheumatoid arthritis, chronic liver and kidney diseases, fracture, infection, peritonitis, pancreatitis, pelvic inflammatory disease, a recent acute coronary syndrome (＜3months), cancer, leukocytosis (＞12.000/µl), leukopenia (＜3.500/µl), Cushing syndrome (with DXAmethasone suppression tests), fever, and medical treatment with anticoagulant  and GC drugs. Patients receiving hormone replacement therapy and antiepileptic medication were also excluded. |
| Zeynel 2013[35] | A total of 1635 older adults who were referred to the outpatient clinic were included in this cross-sectional study. DXA examination can be conducted and peripheral blood samples can be collected. | Those patients, on dialysis, with malign disease, severe liver failure, vitamin B12 deficiency, active infection disease and secondary osteoporosis were excluded from the study. |

Note: NA, not available; DXA, dualenergy X-ray absorptiometry; BMD, Bone Mineral Density; OP, Osteoporosis; RA, Rheumatoid arthritis; eGFR, estimated Glomerular Filtration Rate;

CKD, chronic kidney disease; PTH, parathyroid hormone; KDIGO, Kidney Disease: In the Improving Global Outcomes; T1D, Type 1 Diabetes; T2D, Type 2 Diabetes; DM, diabetes mellitus;

MHT, menopausal hormone therapy; GC, glucocorticoid; NLR, neutrophil-to-lymphocyte ratio; PLR, platelet-to-lymphocyte ratio; MLR, monocyte-to-lymphocyte ratio; SII, systemic immune-inflammation index.
